# Advances in bronchoscopic optical coherence tomography and confocal laser endomicroscopy in pulmonary diseases

**DOI:** 10.1097/MCP.0000000000000929

**Published:** 2022-11-16

**Authors:** Tess Kramer, Pieta C. Wijsman, Kirsten A. Kalverda, Peter I. Bonta, Jouke T. Annema

**Affiliations:** Amsterdam UMC, University of Amsterdam, Department of Pulmonary Medicine, Meibergdreef 9, Amsterdam, The Netherlands

**Keywords:** bronchoscopy, confocal laser endomicroscopy, diagnostics, imaging, optical coherence tomography

## Abstract

Imaging techniques play a crucial role in the diagnostic work-up of pulmonary diseases but generally lack detailed information on a microscopic level. Optical coherence tomography (OCT) and confocal laser endomicroscopy (CLE) are imaging techniques which provide microscopic images *in vivo* during bronchoscopy. The purpose of this review is to describe recent advancements in the use of bronchoscopic OCT- and CLE-imaging in pulmonary medicine.

**Recent findings:**

In recent years, OCT- and CLE-imaging have been evaluated in a wide variety of pulmonary diseases and demonstrated to be complementary to bronchoscopy for real-time, near-histological imaging. Several pulmonary compartments were visualized and characteristic patterns for disease were identified. In thoracic malignancy, OCT- and CLE-imaging can provide characterization of malignant tissue with the ability to identify the optimal sampling area. In interstitial lung disease (ILD), fibrotic patterns were detected by both (PS-) OCT and CLE, complementary to current HRCT-imaging. For obstructive lung diseases, (PS-) OCT enables to detect airway wall structures and remodelling, including changes in the airway smooth muscle and extracellular matrix.

**Summary:**

Bronchoscopic OCT- and CLE-imaging allow high resolution imaging of airways, lung parenchyma, pleura, lung tumours and mediastinal lymph nodes. Although investigational at the moment, promising clinical applications are on the horizon.

## INTRODUCTION

Imaging techniques such as (high-resolution) computed tomography (HRCT), X-ray, trans-thoracic ultrasound, PET and MRI are cornerstones in diagnosing a wide variety of pulmonary diseases [1]. Although these techniques provide important clinical insights, they are limited in resolution and unable to provide near-histological information. Therefore, regularly additional tissue sampling is needed for pathologic evaluation to establish a precise diagnosis. Although tissue biopsies are often regarded as the gold standard, they are invasive, only provide focal information of sampling location and are subject to sampling errors.

Optical coherence tomography (OCT) and confocal laser endomicroscopy (CLE) are imaging techniques compatible with conventional bronchoscopy for (near-)microscopic scanning. Several studies have evaluated OCT- and CLE-imaging of the airway wall, lung tumours, lung parenchyma, mediastinal lymph nodes and pleura to improve the diagnosis and understanding of pulmonary diseases [2]. In 2017, Wijmans *et al.*[3] published a review article about OCT- and CLE-imaging in pulmonary diseases in this journal. In the last 5 years, new developments have been reported including potential clinical applications. The aim of this review article is to provide an update on OCT- and CLE-imaging in pulmonary medicine and to discuss future clinical perspectives. 

**Box 1:**
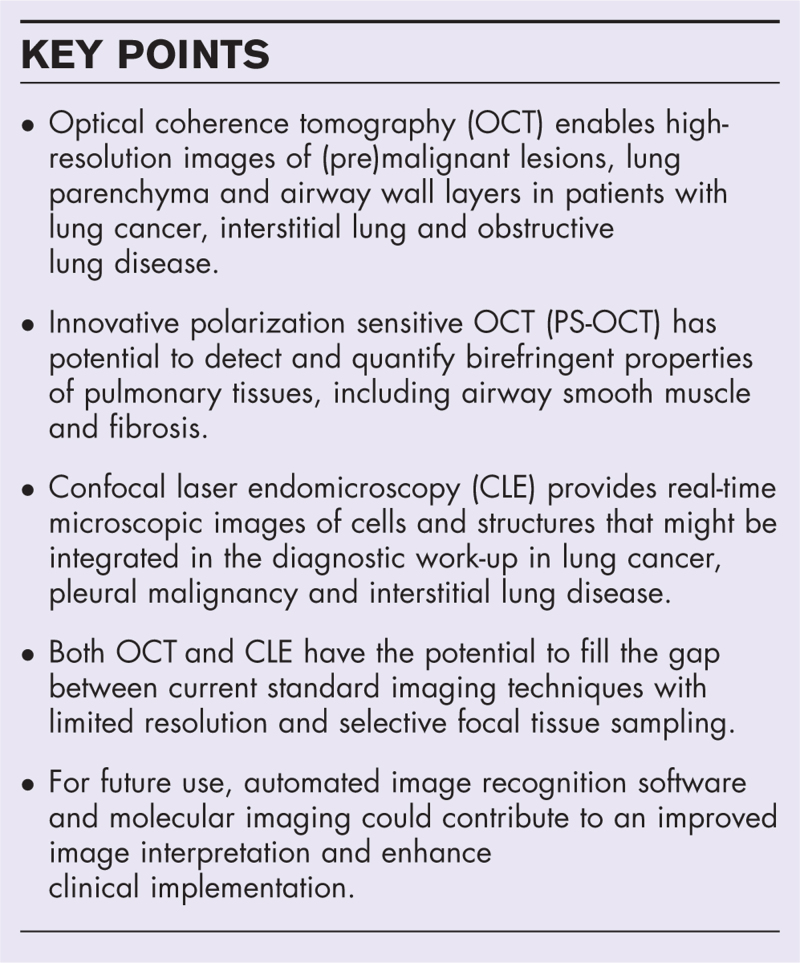
no caption available

## OPTICAL COHERENCE TOMOGRAPHY

### Technical background

**FIGURE 1:**
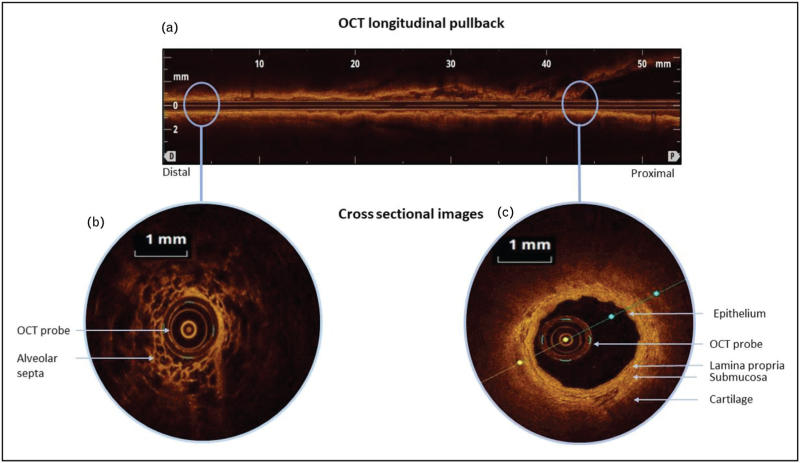
Bronchoscopic OCT-imaging imaging of the lung parenchyma and airways in an asthma patient. (a) Bronchoscopic OCT longitudinal pullback (54 mm) in an asthma patient from distal (alveolar compartment) to proximal (airway compartment), with cross-sectional images of lung parenchyma with alveoli (b) and airway with its wall layer structures (c).

OCT is a near-infrared light-based imaging technique, which obtains high resolution images from tissues of interest [4]. As there are variances in time of light backscattering from different tissue structures, OCT is able to generate images with a resolution of ±10 μm and imaging depth of 2–3 mm [5]. In clinical practice, OCT has been used in ophthalmology to assess the retina and in vascular disease to assess chronic thromboembolic pulmonary hypertension, coronary artery stenosis and stent placement [6–9]. In pulmonology, this imaging technique has been applied in several pulmonary diseases, but so far in research settings only [2]. During bronchoscopy, an OCT probe is inserted through the working channel of the bronchoscope and real-time imaging of the lung parenchyma and airways is performed by generating pullbacks from distal to proximal (Fig. 1). Circumferential two-dimensional images can be reconstructed into a three-dimensional overview. A different approach is OCT-imaging through a biopsy needle (needle-based OCT) to guide needle tissue sampling. The latest advancement includes polarization sensitive OCT (PS-OCT), which enables the detection of pulmonary tissues with birefringence properties [10,11^▪^]. This review summarizes OCT results in malignancy, interstitial and obstructive lung diseases.

### Lung cancer

In 2005, the first report on bronchoscopic OCT-imaging in five patients with endobronchial lung tumours was published. OCT-imaging of the tumour area found dispersed distribution of high backscattering areas and loss of the normal airway wall layer architecture (Table 1) [12]. Thereafter, OCT-imaging of endobronchial lesions has demonstrated to provide additional value in the identification and characterization of premalignant bronchial lesions as compared to white-light evaluation [13,14]. Furthermore, Hariri *et al*. [15] performed ex-vivo OCT-imaging on 82 fresh lung tumour specimens and reported distinguishing criteria for primary lung adenocarcinoma, squamous cell carcinoma and poorly differentiated carcinoma. Although encouraging, the authors concluded that OCT-imaging cannot replace histological evaluation to distinguish different tumour types [15]. A potential application of OCT-imaging in lung cancer diagnosis, might be to provide feedback on the optimal biopsy needle positioning [16–19]. For this purpose, several needle-based OCT catheters have been investigated by multiple groups [18,20–25]. Hariri *et al*. [17] performed needle-based OCT-imaging in 26 thoracic lymph nodes and identified malignant lymph nodes with irregular architecture and variations in signal intensity and attenuation (Table 1). In addition, necrosis was identified as nested structures with a signal-intense periphery and central signal-poor homogeneous tissue [17]. Furthermore, in ex-vivo experiments, PS-OCT-imaging was able to accurately differentiate tumours with high from low fibrosis content, which can potentially impact diagnostic yield [26,27]. To date, no study has evaluated the use of needle based and PS-OCT-imaging in lung cancer patients *in vivo*, which deserves further studies.

**Table 1:** Bronchoscopic optical coherence tomography imaging in lung cancer, interstitial lung disease and obstructive lung disease

	Application	Identified patterns	Clinical perspectives	Stage of innovation^a^
Lung cancer	Identification of malignant lesions using OCT- imaging [13–17]	Endobronchial tumour:- high backscattering areas and loss of layered structure- ragged, irregular, dark line between light areas in the sub-epithelium	Endobronchial (pre) malignancy) detectionMolecular OCT-imaging for tumour detection	Development (2A)
	Needle based OCT in lymph nodes (ex-vivo) [18–25]	Normal lymph nodes:- round structure, homogeneous with moderate signal intensity and minimal architectural variationMalignant lymph nodes:- irregular architecture and variation in signal intensity and attenuation	Needle based malignancy detection in-vivoMolecular OCT-imaging for tumour detection	Proof of concept (1)
	Identification of malignant lesions using PS-OCT imaging (ex-vivo) [26,27]	Normal lung parenchyma:- lattice like alveolar structure- moderate birefringenceTumour area with low fibrosis:- low birefringenceTumour area with high fibrosis:- high birefringence	Lung cancer detection in-vivoIdentification of tumour areas with high versus low fibrosis for intra-procedural tumour yield assessment	Proof of concept (1)
Interstitial lung disease	OCT-imaging of the lung parenchyma [30,31^▪▪^]	Normal lung parenchyma:- thin, lattice-like alveoli and evenly sized.Fibrotic lung parenchyma:- Loss aeration- Microscopic honeycombing and traction bronchiectasis- irregularly shaped, with thickened alveoli	Diagnosing ILD (UIP versus non-UIP)ILD assessment and treatment effect evaluation	Development (2A)
	Identification of fibrotic tissue using PS-OCT imaging [32^▪^,33^▪^]	Normal lung parenchyma:- thin, lattice-like alveoli with very little birefringence present.Destructive fibrosis:- tissue with destruction of underlying lung architecture with increased birefringence presenceNondestructive interstitial fibrosis:- thickening of alveolar walls with increased presence birefringence	Early and progressive fibrosis detectionDifferentiation of fibrotic tissue and other cause for loss of aerated lung tissueILD assessment and treatment effect evaluation	Development (2A)
Obstructive lung disease	Identification airway wall layers using OCT- imaging [35,36,40^▪^]	Airway wall layers and structures:- epithelium and basement membrane- lamina propria- submucosa- cartilage- collagen and elastin (ex-vivo)	Airway remodelling detectionEvaluation of treatments targeting airway remodellingMolecular OCT-imaging for airway remodelling detection	Development (2A)
	Quantification of airway dimensions [37,38,39^▪^]	Dynamic changes in:- airway lumen- airway wall thickness	Evaluation of airway remodelling	Development (2A)
	Identification airway wall layers using PS-OCT imaging [11^▪^,^35^]	Airway wall layers- airway smooth muscle visualisation and quantification- cartilage	Detection of extra cellular matrix componentsMolecular OCT-imaging for airway remodelling detection	Development (2A)

a Stage of innovation based on IDEAL guidelines for surgical interventions: Stage 1 Proof of concept, Stage 2a Development, Stage 2b Exploration, Stage 3 Assessment, Stage 4 Surveillance [69].

### Interstitial lung diseases

**FIGURE 2:**
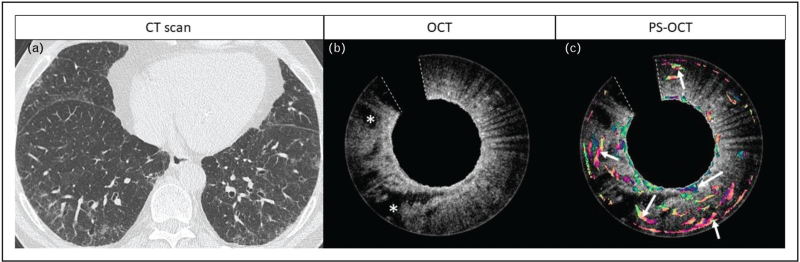
Chest CT, bronchoscopic conventional OCT and PS-OCT images of an ILD patient. (a) High-resolution chest CT (HR-CT) scan with unclassifiable ILD. (b) Conventional bronchoscopic OCT performed in the right lower lobe showing a cross-sectional image visualizing the alveolar compartment with loss of normal alveolar architecture and enlarged alveolar spaces(∗). (c) Corresponding bronchoscopic PS-OCT cross-sectional image showing birefringence (arrows) in these areas indicating fibrosis. The white dotted lines in OCT and PS-OCT images delineate the tissue area not optically accessible because of wires to the motor.

Diagnosing ILD is challenging and currently no gold standard single test is available to diagnose and differentiate different subtypes of ILD [28]. Histological evaluation by surgical lung biopsies or bronchoscopic cryobiopsies may be indicated, but are invasive, associated with significant mobridity and are not useful for (longitudinal) measurements [29]. Hence, minimally invasive bronchoscopic OCT-imaging could be of added value. The alveolar compartment can be visualized in high detail by OCT-imaging, allowing the detection of microscopic honeycombing undetectable by HRCT [30]. Nandy *et al*. [31^▪▪^] demonstrated that OCT can differentiate between an usual interstitial pneumonia (UIP) and a non-UIP histopathological pattern in fibrotic lung disease. In this study, 27 patients were evaluated and OCT outcomes were compared with histology from surgical lung biopsies, demonstrating a 100% sensitivity and specificity for diagnosing a UIP histopathological pattern (Table 1). The potential to differentiate between UIP and non-UIP by adding OCT to standard bronchoscopic bronchoalveolar lavage might impact the diagnostic workup of patients with ILD and may overcome need for surgical or cryobiopsies in selected cases. Although conventional OCT has successfully identified anatomical structures and characteristics compatible with subsets of ILD, PS-OCT enhances the ability to visualize fibrosis by detecting birefringence tissue properties (Fig. 2). Two study groups are currently working on endobronchial PS-OCT (EB-PS-OCT) in the field of fibrotic ILD. Recently, Nandy *et al.*[32^▪^] reported the feasibility of EB-PS-OCT for microscopic assessment of fibrosis. Furthermore, a recent abstract reported the possibility to quantify the amount of fibrosis by birefringence structures using histology as the reference standard [33^▪^]. Whether EB-PS-OCT allows improved regional identification of (early) fibrotic tissue in comparison to standard HRCT imaging, is currently under investigation.

### Obstructive lung disease

Airway remodelling is a pathological feature of obstructive lung disease in asthma and chronic obstructive pulmonary disease (COPD). Airway remodelling is characterized by changes in the airway wall including alterations in the airway smooth muscle (ASM) and extracellular matrix (ECM) [34]. The current imaging standard for airway remodelling detection is HRCT, which has limited resolution and coincides with radiation exposure. Alternatively, histological evaluation of airway biopsies can be used to assess airway remodelling, but these are invasive and provide only focal airway wall information. OCT has the potential to overcome these limitations and several study groups have reported on OCT for this clinical purpose (Table 1). First, conventional OCT-imaging was compared with HRCT to identify and quantify airway wall layers [35,36] and dimensions in both asthma and COPD [37,38]. Recently, Su *et al.*[39^▪^] reported airway lumen diameter changes measured by OCT after the application of bronchodilators in asthmatic patients in airways from the third till the ninth generation. Apart from these dynamic changes, OCT has the ability to directly visualize and quantify tissue compartments by the use of deep learning assistance *in vivo*[40^▪^] and is able to visualize specific ECM components by using a threshold technique on light scattering intensities (Fig. 1). In an ex-vivo study, the potential of this strategy to automatically identify and quantify ECM structures in the airway wall was demonstrated [41].

**FIGURE 3:**
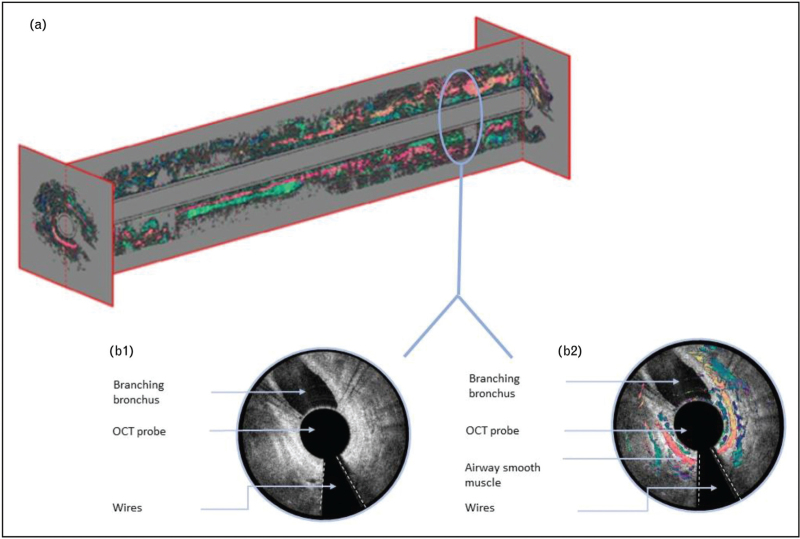
Bronchoscopic PS-OCT pullback with proximal cross-sectional images of an airway in an asthma patient. (a) Bronchoscopic PS-OCT reconstruction of a longitudinal pullback from distal to proximal in an airway of an asthma patient with B1) a proximal cross-sectional standard OCT image of the airway showing airway wall layers and B2) a corresponding PS-OCT image incorporating optic axis orientation detection of the tissue enabling visualization of airway smooth muscle. The white dotted lines in OCT and PS-OCT images delineate the tissue area not optically accessible because of wires to the motor.

Next to conventional OCT, PS-OCT has shown promising results in visualization and quantification of ASM in the airways (Fig. 3) which is considered a key structure in bronchoconstriction and release of inflammatory mediators in asthma patients [32^▪^]. Moreover, two studies measured the ASM content after bronchial thermoplasty, an endoscopic treatment for severe asthma to decrease the ASM [11^▪^,^38^]. Taken together, (PS)-OCT enables near-histological investigation of complete airways and as such has the potential to assess changes in airway remodelling and might improve the understanding of the pathogenesis and treatment effects in obstructive lung diseases (Table 1).

For future research purposes, the combination of (PS-)OCT with the detection of fluorescently labelled antibodies (immune-OCT), for example biologicals used in asthma treatment, might be developed to elucidate cellular targets of the biologicals and the treatment effects on airway remodelling [42].

## CONFOCAL LASER ENDOMICROSCOPY

### Technical background

CLE is a laser-based imaging technique that allows the visualization of cells *in vivo* for a real-time microscopic evaluation of the tissue investigated. With an objective lens, the laser-light (most commonly 488 nm) is focused on the tissue wherein it strikes an autofluorescent structure (e.g. elastin) or fluorescent dye (e.g. fluorescein). Light is emitted back to the detector using a pin-hole to acquire high-resolution images with a resolution up to 3.5 μm, maximum imaging depth of 70 μm and maximum field of view of 600 μm [2,3]. In the field of gastro-enterology, CLE-imaging has been used in clinical practice for the diagnosis of Barret's disease and pancreatic cysts [43–46]. In pulmonology, CLE-imaging is mainly investigational and further research is ongoing before implementation in clinical practice.

There are two different applications of CLE-imaging, namely probe-based CLE-imaging (pCLE), wherein the flexible CLE-probe is inserted through the working channel of the bronchoscope, and needle-based CLE-imaging (nCLE), wherein the probe is advanced through the lumen of a biopsy needle. The pCLE technique is frequently used for visualizing the autofluorescent elastin fibres of the alveoli and airways [47]. Although there are different CLE-probes, the Alveoflex CLE-probe (Mauna Kea Technologies, Paris, France) has the largest field of view (optic area of 1.13 mm^2^ and image plane 50 μm) and is therefore most commonly used for pCLE-imaging.

nCLE-imaging is most commonly used for the evaluation of cellular structures in mediastinal lymph nodes and lung lesions [48^▪▪^,^49^]. The AQ-flex CLE probe (Mauna Kea Technologies) is usually used for nCLE-imaging, as this smaller probe fits through the lumen of a biopsy needle. The AQ-flex probe has a smaller field of view with an optic area of 0.33 mm^2^ and an image plane of 20 μm. As (tumour-)cells do not have autofluorescent properties, it is recommended to administer fluorescein intravenously to create a contrast between the cells and bright fluorescein-rich stromal background. In this review, we aim to describe current advancements of CLE-imaging in lung cancer, pleural malignancy and ILD.

### Lung cancer

Initially, CLE-imaging for the diagnosis of lung cancer was performed by scanning the surface of the airways and lung tumours using the pCLE-technique. Although some indirect malignant patterns were identified, no clear delineation of tumour cells was visualized due to the limited penetration depth [50–55]. Another disadvantage of pCLE-imaging is that as the CLE probe occupies the working channel of the bronchoscope, the CLE probe has to be removed before taking a biopsy sample and subsequent biopsies are taken without CLE guidance.

**FIGURE 4:**
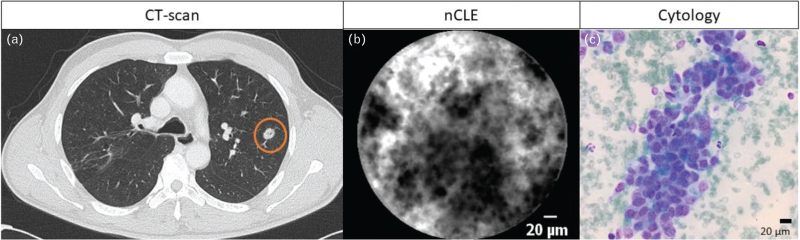
Bronchoscopic nCLE-imaging of a peripheral lung tumour. (a) Chest CT-scan with a lung tumour in the left lower lobe (circle). (b) nCLE-imaging of the lung lesion shows clusters of enlarged pleomorphic tumour cells. (c) Corresponding cytology representing a pulmonary metastasis of a urothelial carcinoma.

With the development of the smaller AQ-flex probe, nCLE-imaging at the biopsy needle tip was introduced. Wijmans *et al.*[49,56] performed for the first time nCLE-imaging in central lung tumours and metastatic lymph nodes during endoscopic ultrasound procedures. Three nCLE criteria for lung cancer were identified (enlarged pleomorphic cells, dark clumps and directional streaming) and validated with 90% accuracy [49]. In a subsequent study, bronchoscopic nCLE-imaging was performed in 24 peripheral lung tumours (Fig. 4). The previous identified nCLE malignancy criteria were confirmed and novel criteria for airway and lung parenchyma (elastin fibres, alveoli, bronchial epithelium and still image) were identified (Table 2). Therefore, the authors hypothesized that nCLE-imaging can be used to fine-tune the optimal needle positioning [48^▪▪^]. In a recent case report, this hypothesis was tested, and nCLE-imaging was combined with robotic bronchoscopy in a small, partially cystic lesion to determine the exact sampling location [57^▪^]. Whether nCLE-imaging in suspected lung lesions will eventually result in an improved diagnostic yield is under investigation. In a future concept, immediate tumour ablation can be performed after malignancy confirmation. Molecular nCLE-imaging with fluorescent malignant tracers can contribute to this concept. Recently, a study performed *in vitro* and *ex vivo* near-infrared CLE-imaging using a malignant molecular tracer. Blinded raters were able to accurately distinguish malignant tissue from healthy tissue [58^▪▪^,59^▪^]. Whether similar results can be achieved *in vivo,* including benign disease differentiation, should be evaluated.

**Table 2:** Bronchoscopic confocal laser endomicroscopy imaging in lung cancer, pleural malignancy and interstitial lung disease

	Application	Identified patterns	Clinical perspectives	Stage of innovation^a^
Lung cancer	Identification of malignant lesions using needle-based CLE-imaging (nCLE) [47,48^▪▪^,^55^,^56^]	Malignancy:- enlarged pleomorphic cells- dark clumps- directional streamingNormal airway/lung parenchyma:- elastin fibres- alveoli- still image- bronchial epithelium	Improve diagnostic yield by fine-tuning of sampling areaMolecular CLE-imaging for in-vivo analysis of different tumour types	Development (2A)
	Identification of malignant lesions using probe-based CLE-imaging (pCLE) [50–55]	Malignancy:- black holes- elastin disorganization- cellular infiltrates	Identification of (pre)malignant endobronchial lesions	Development (2A)
Pleural malignancy	Identify malignant pleural disease during thoracoscopic procedures using probe-based CLE-imaging (pCLE) [60–63]	Malignant pleura:- enlarged pleomorphic cells- dysplastic vessels- abnormal architectureBenign pleura:- full chiaseed sign- cell shape homogeneity	Improve thoracoscopic diagnostic yield by identifying optimal sampling area	Exploration (2B)
	Identification of malignant cells in pleural effusion using probe-based CLE-imaging (pCLE) [62^▪^]	Malignant effusion:- variated cell size- enlarged nucleus- irregular nucleus and cell membranes	Improve pleural fluid malignancy detection	Proof of concept (1)
Interstitial lung disease	Identify ILD characteristics by probe-based CLE-imaging (pCLE) of the lung parenchyma [64,65,67–69]	Cellular ILD:- hypercellular patternsFibrotic ILD:- disorganized elastin network- decreased alveolar openings- thickened septal fibres	Discriminate cellular ILD from fibrotic ILDFibrosis detection	Development (2A)
	Assess optimal sampling area with probe-based CLE-guided (pCLE) cryobiopsies of the lung parenchyma [66]	Mild fibrosis:- mild increase of elastin fibres- preserved alveolar architectureDense fibrosis:- dramatic increase of elastin fibres- destruction alveolar architecturePleura:- dense cross-fibre pattern	Improve diagnostic yield of cryobiopsies in ILD patientsReduce pneumothorax complication rate	Development (2A)

a Stage of innovation based on IDEAL guidelines for surgical interventions: Stage 1 Proof of concept, Stage 2a Development, Stage 2b Exploration, Stage 3 Assessment, Stage 4 Surveillance [69].

### Pleural malignancy

Several studies performed CLE-imaging for the diagnosis of pleural malignancies [60–63]. One study performed both pCLE and nCLE-imaging of the pleura during 20 diagnostic procedures, including thoracoscopy, surgical excision, ultrasound and endoscopic ultrasound [61]. In comparison with 105 pleural biopsies, distinctive CLE patterns for pleural tumour deposits, malignant infiltration in fat tissue and benign fibrotic pleural tissue were validated with moderate interobserver agreement [61]. The most extensive experience, however, was gained recently by performing pCLE-imaging during thoracoscopic procedures [62^▪^]. Of the 62 included patients, 36 had benign and 26 had malignant pleural disease upon histological evaluation. On the basis of 310 CLE-videos, the criteria ‘abnormal tissue architecture with pleomorphic cells’ and ‘dysplastic vessels’ were significantly associated with malignancy, whereas the criteria ‘full chia seeds sign’ and ‘cell shape homogeneity’ were associated with benign pleura (Table 2). Whether CLE-guided pleura sampling will result in an improved diagnostic yield should be evaluated.

One ex-vivo study performed pCLE-imaging in pleural effusions and found high sensitivity and specificity for the detection of enlarged pleomorphic cells in cytological malignant pleural effusions (Table 2) [63]. However, as cytology of pleural effusions is associated with a sensitivity of approximately 60% [63], the added value of CLE-imaging in pleural effusions is unknown.

### Interstitial lung disease

**FIGURE 5:**
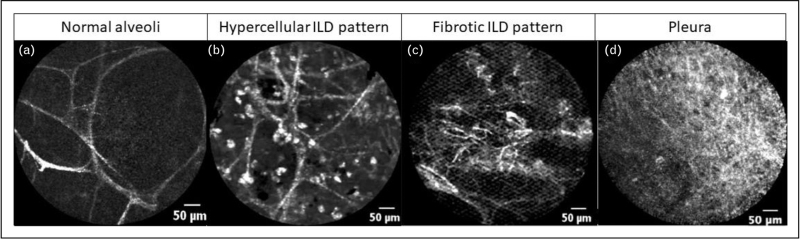
Bronchoscopic CLE-imaging in ILD. (a) CLE-imaging of normal lung parenchyma with thin alveolar septae and wide alveolar openings. (b) CLE-imaging in a patient with desquamitive ILD shows filling of the alveolar spave with autofluorescent cells. (c) CLE-imaging in a patient with fibrotic nonspecific idioptahic pneumonia demonstrating an increase of elastin fibres with destruction of alveolar architecture. (d) CLE-imaging of the pleura showing a dense cross fibre pattern.

As ILD is a disease affecting the lung parenchyma, several studies have evaluated CLE-imaging of the alveolar compartment to improve the diagnosis of ILD [64–66]. Initial experience was gained by performing pCLE-imaging in patients with amiodarone related pneumonia, pulmonary alveolar proteinosis and alveolar microlithiasis. It was demonstrated that autofluorescent macrophages, calcispherites and complexes of granulated lipoproteinaceous substances can be identified on pCLE-imaging [67–69]. Meng *et al*. [65] performed pCLE-imaging in 27 patients with different subtypes of ILD and showed a significant correlation between hypercellular patterns on pCLE-imaging and inflammatory patterns on CT-scan (Table 2). Surprisingly, however, no significant correlation between densely packed elastin fibres on pCLE-imaging and fibrosing interstitial pneumonia was found [65]. In a subsequent study, Salaün *et al*. [64] performed pCLE-imaging in 80 people, including 59 ILD patients and 21 healthy volunteers. Of the 14 pCLE patterns initially described, nine patterns were significantly more frequent in at least one of the ILD groups compared with healthy volunteers (Table 2). This included disorganized, dense septal alveolar fibres in patients with idiopathic pulmonary fibrosis [64]. Furthermore, Wijmans *et al.*[66] suggested the use of pCLE-imaging to guide transbronchial cryobiopsies to improve the diagnostic yield by differentiating normal from diseased lung parenchyma (Fig. 5). This application might also reduce the current pneumothorax complication rate by real-time identification of the conducting airways, parenchyma and pleura (Table 2) [66]. Whether CLE-imaging will actually improve the diagnostic yield and reduce complication rates, should be the subject of prospective comparative studies.

## DISCUSSION

Both bronchoscopic OCT- and CLE-imaging have been applied for real-time, near-microscopic evaluation *in vivo* in a wide variety of pulmonary diseases and different anatomical compartments, including lung cancer, pleural malignancy, interstitial and obstructive lung diseases. For clinical application, clinical perspectives for (PS-)OCT imaging include assessment of airway remodelling in obstructive airway disease and differentiating (fibrotic) subtypes of ILD. CLE-imaging has shown promising results to improve the diagnosis of lung cancer and pleural malignancy. In a desirable future concept, immediate tumour ablation could be performed after (nCLE) malignancy confirmation. Molecular OCT- and CLE-imaging with labelled markers will be of great interest to assess tumour environment and the distribution of antibodies to evaluate treatment effects in pulmonary diseases. Furthermore, the development of automated image recognition software could contribute to an improved image interpretation and ease clinical application. However, as most studies are still exploratory and performed in research settings, larger and comparative studies are needed before implementation in standard clinical care.

## Acknowledgements

*The authors would like to thank M. Vaselli and T. Soldati for providing PS-OCT images and L. Wijmans for providing CLE images of fibrotic pulmonary tissue.*

### Financial support and sponsorship

*This review is author-initiated without industrial support. During the conduct of clinical studies, we received financial and material support for study purposes from Mauna Kea Technologies and Abbot (Abbott acquired St. Jude Medical). Furthermore, we received financial support from MedPhot (research program 00770372 with project number 00770384), which is partly financed by the Dutch Research Council (NWO).*

### Conflicts of interest

*There are no conflicts of interest.*

## REFERENCES AND RECOMMENDED READING

Papers of particular interest, published within the annual period of review, have been highlighted as:

▪ of special interest
▪▪ of outstanding interest
